# Pediatric-Onset Relapsing Polychondritis With Otolaryngeal Manifestations

**DOI:** 10.7759/cureus.40085

**Published:** 2023-06-07

**Authors:** Nicholas J Figaro, Keegan A Figaro, Jibran S Juman, Rodolfo Arozarena, Keisha Davis King, Solaiman Juman

**Affiliations:** 1 Otolaryngology-Head and Neck Surgery, Eric Williams Medical Sciences Complex, Champ Fleurs, TTO; 2 Medicine, Eric Williams Medical Sciences Complex, Champ Fleurs, TTO; 3 Otolaryngology-Head and Neck Surgery, The University of the West Indies, Saint Augustine, TTO; 4 Rheumatology, Eric Williams Medical Sciences Complex, Champ Fleurs, TTO

**Keywords:** conjunctivitis, subglottic stenosis, saddle nose deformity, auricular chondritis, relapsing polychondritis, pediatric

## Abstract

Relapsing polychondritis (RP) is a rare autoimmune disease that can present with various clinical manifestations. Among the affected sites, the ear, nose, and throat cartilages are frequently involved, often leading to subtle and episodic symptoms that can be challenging to diagnose. A high index of suspicion is necessary for the early identification of these subtle signs, which can aid in early diagnosis and prompt management. In this report, we present a rare case of pediatric-onset relapsing polychondritis that was initially misdiagnosed as laryngotracheobronchitis.

## Introduction

First described in 1923 by Jaksch-Wartenhorst as “polychondropathia,” relapsing polychondritis (RP) is an extremely rare multisystemic autoimmune disorder characterized by widespread, destructive, inflammatory lesions of cartilage [1]. The disease classically causes chondritis of the ear, nose, and larynx and progresses through recurrent flares leading to floppy ears, saddle nose deformity, and laryngotracheal stenosis. The insidious onset and variable, episodic expression of clinical features over time often make the diagnosis of RP difficult to establish [2]. Approximately one-third of RP patients will be associated with another autoimmune disorder [3].

The episodic nature of RP makes treatment difficult; several immunosuppressive medications, including steroid and steroid-sparing disease-modifying antirheumatic drugs, have been used with varying success [4]. Here, we report a rare case of pediatric-onset relapsing polychondritis, which had been misdiagnosed as laryngotracheobronchitis.

## Case presentation

**Figure 1:**
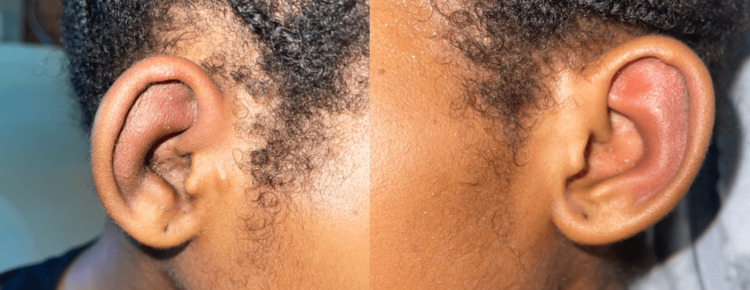
Bilateral auricular chondritis

**Figure 2:**
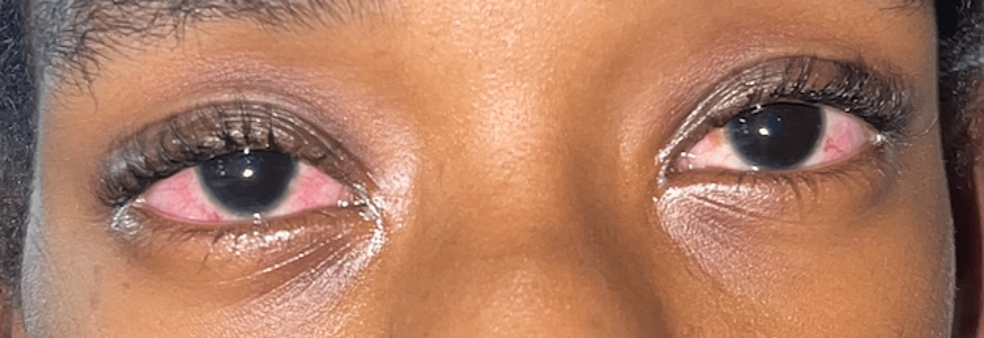
Bilateral conjunctivitis

**Figure 3:**
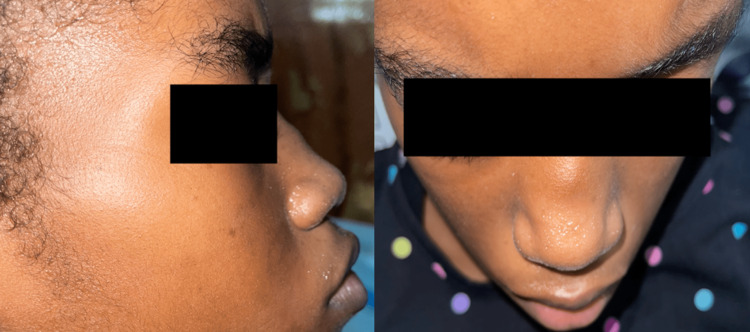
Saddle nose deformity

**Figure 4:**
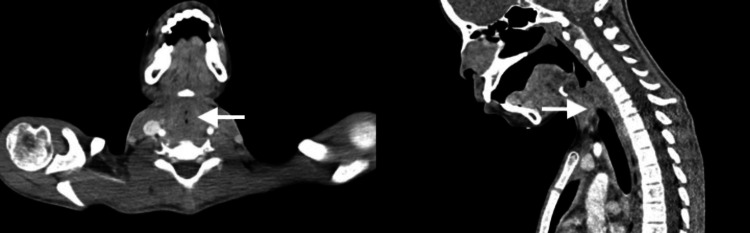
Axial and sagittal computed tomography images showing subglottic stenosis

**Figure 5:**
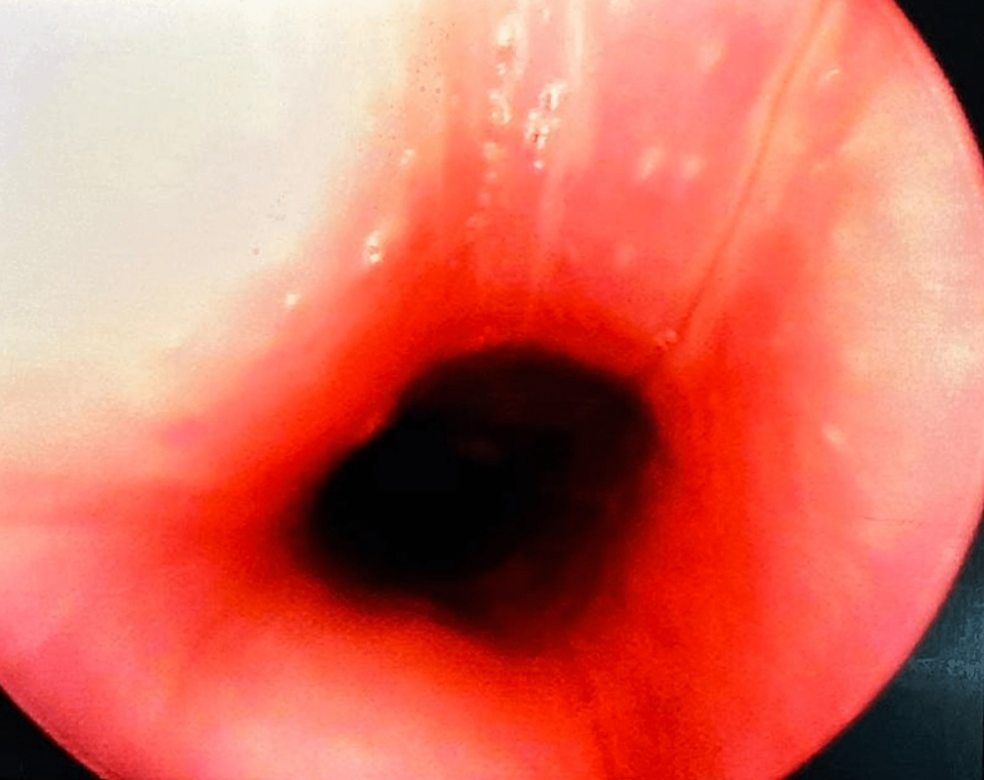
Microlaryngobronchoscopy showing subglottic narrowing

A 12-year-old Afro-Trinidadian female presented to the pediatric emergency department with a fever and a persistent nonproductive cough. The patient was diagnosed with laryngotracheobronchitis; managed with intravenous antibiotics, steroids, and nebulized epinephrine; and subsequently discharged. She re-presented on three subsequent occasions with similar symptoms, where she was treated and discharged. Six weeks later, she was readmitted with fever, red eyes, and hoarseness of voice; as a result, an urgent otolaryngology review was requested. Her vital signs were as follows: body temperature of 38.5°C, heart rate of 130 beats per minute, blood pressure of 115/75 mm Hg, respiratory rate of 32 breaths per minute, and oxygen saturation level of 96% on room air. A physical examination of the patient revealed loud biphasic stridor and labored breathing. The patient’s pinna was tender and erythematous with sparing of the lobule (Figure 1); she had bilateral conjunctivitis (Figure 2) and a saddle nose deformity (Figure 3). Flexible nasal endoscopy showed erythematous vocal cords with normal vocal cord movement. Laboratory findings showed a hemoglobin of 10.3 g/dL, a white blood cell count of 14,500/mm^3^, an elevated erythrocyte sedimentation rate of 160 mm/hour, and a C-reactive protein of 350 mg/dL. Other paraclinical parameters (urinalysis and thyroid, renal, and liver function tests) were within normal limits. The patient’s data was reviewed by the pediatric intensivist, and the patient was started on high-flow supplemental oxygen, nebulized epinephrine, intravenous steroids, and antibiotics. A computed tomography scan of the patient’s neck and chest demonstrated a 2 cm-narrowed subglottic segment (Figure 4) and a diffuse thickening and luminal narrowing of several segmental bronchioles in keeping with bronchiolitis, respectively. Despite medical management, the patient developed acute respiratory failure. The patient was taken to the operating theater for intubation; however, multiple attempts by a senior pediatric anesthetist failed, and an awake tracheostomy and microlaryngobronchoscopy were performed. The microlaryngobronchoscopy confirmed significant subglottic narrowing (Figure 5).

Immunological tests for antinuclear antibodies, double-stranded DNA, antiphospholipid antibodies, antineutrophil cytoplasmic antibodies, anti-cyclic citrullinated peptide antibodies, and rheumatoid factor were negative. Using McAdam’s criteria (Table 1) [5], the patient was clinically diagnosed with relapsing polychondritis. After reviewing the patient’s data, the rheumatologist agreed with the clinical diagnosis. She was started on 50 mg of oral prednisolone daily and 10 mg/week of methotrexate. The patient had an uneventful one-week stay at the pediatric intensive care unit, before being transferred to the ward. Her symptoms and inflammatory markers considerably decreased after three weeks on the ward (erythrocyte sedimentation rate of 45 mm/hour and C-reactive protein of 42 mg/dL). After that, she was discharged from the hospital with a tapering prednisolone dose of 15 mg daily and a methotrexate dose of 8 mg weekly. At her nine-month outpatient follow-up, despite being clinically stable and continuing to gradually wean off of her prednisolone regimen, the patient is nonetheless dependent on a tracheostomy and continues to receive 8 mg/week of methotrexate. In the interim, an international quaternary center with advanced airway expertise has been contacted to review and possibly address the patient’s subglottic stenosis.

**Table 1:** Diagnostic criteria for relapsing polychondritis

McAdam’s criteria	Clinical features
The presence of three or more of the given clinical features	Recurrent chondritis of both auricles
	Nonerosive inflammatory arthritis
	Chondritis of the nasal cartilages
	Ocular inflammation including conjunctivitis, scleritis, episcleritis, conjunctivitis, and/or uveitis
	Chondritis of the respiratory tract involving the laryngeal and/or tracheal cartilages
	Cochlear and/or vestibular damage manifested by sensorineural hearing loss, tinnitus, and/or vertigo

## Discussion

Relapsing polychondritis is an uncommon but progressive autoimmune condition that affects the body’s cartilage and other connective tissues and can result in fatal complications [6]. The disease is characterized by recurring episodes of cartilage inflammation and degeneration in the joints, larynx, trachea, ears, and nose [7]. Vasculitis, myocarditis, ocular inflammation, audiovestibular dysfunction, and nonerosive arthritis are other clinical characteristics [3]. Relapsing polychondritis is thought to affect 3.5 per million people, with a median age of onset between the fourth and fifth decade of life [5]. However, RP can affect anyone at any age, with juvenile RP accounting for less than 5% of recorded instances [8,9]. Although the precise pathophysiology of RP is not yet known, it is believed to be an autoimmune reaction to type 2 collagen [10].

Due to the rarity of RP, many medical professionals are not familiar with its signs or diagnostic standards. Relapsing polychondritis frequently manifests as a variety of heterogeneous, albeit seemingly unrelated, signs and symptoms, which often pose a major diagnostic dilemma [2,3]. The patient presented to the hospital on multiple occasions before receiving a conclusive diagnosis in the index case, which illustrates this diagnostic challenge.

Relapsing polychondritis is diagnosed clinically because there are no specific laboratory tests, histological patterns, or imaging tests for it [2,8]. Classically, six diagnostic criteria of RP have been established by McAdam et al. [5], which are summarized in Table 1. The diagnosis of RP is made by the presence of three or more of these clinical criteria. Later, Damiani and Levine [2] modified these criteria by including the presence of at least one McAdam’s criteria and positive histological confirmation or two McAdam’s criteria and a favorable response to corticosteroid or dapsone treatment. In this case, the patient met four of the six McAdam’s criteria for the diagnosis of RP [1,2,11].

The involvement of the laryngotrachea by RP is a significant source of morbidity and mortality and affects approximately 90% of pediatric patients [11]. Laryngeal chondritis typically presents with thyroid cartilage tenderness, nonproductive cough, hoarseness, stridor, and wheezing [1]. Respiratory symptoms are commonly accredited to inflammation, leading to airway narrowing and/or the loss of cartilaginous structural support particularly in the subglottic region and trachea [2]. The resultant airway narrowing may require tracheal dilation, stenting or tracheostomy, and reconstructive surgery [12]. Due to airway inflammation and collapse, coughing is less effective at clearing secretions. According to several reports, between 10% and 50% of individuals with RP die from these respiratory problems, which are primarily tracheal collapse and infections [6,13].

Due to its rarity, no clinical trials have been conducted; as such, there are no evidence-based guidelines for the treatment of RP [5]. The goal of treatment is to control the acute inflammatory crisis and the long-term suppression of the immune-mediated pathogenetic pathways [1]. Generally, corticosteroids are the primary treatment, although immunosuppressive medications such cyclophosphamide, cyclosporine A, azathioprine, methotrexate, and mycophenolate mofetil can be used in individuals with persistent or refractory illness [1,13]. Tumor necrosis factor (TNF) antagonists, interleukin 1 (IL-1) receptor antagonists, anti-IL-6 receptor antibodies, and anti-cluster of differentiation 20 (CD20) monoclonal antibodies are a few biological modifiers that have been used in recent research to ameliorate symptoms with various degrees of success [6,13]. Typically, the prognosis of RP varies and is based on the organs involved and the patient’s reaction to treatment. However, compared to the general population, mortality rates of RP are more than twice as high [3,10].

## Conclusions

RP is a rarely occurring, potentially lethal multisystemic autoimmune condition that typically affects cartilaginous tissues. Due to the wide range of clinical symptoms that patients present with and the low incidence of RP, its diagnosis is frequently made late or not at all in children. Due to the lack of standardized treatment standards, RP therapy is currently empiric and determined by the severity of the organs involved and the activity of the illness. This case demonstrates the importance of being vigilant and exercising critical thinking when treating a patient who has persistent symptoms without a known explanation. Otolaryngologists should have a thorough understanding of this condition in order to help with the diagnosis and treatment of RP-related complications.
